# Abberant α-Synuclein Confers Toxicity to Neurons in Part through Inhibition of Chaperone-Mediated Autophagy

**DOI:** 10.1371/journal.pone.0005515

**Published:** 2009-05-13

**Authors:** Maria Xilouri, Tereza Vogiatzi, Kostas Vekrellis, David Park, Leonidas Stefanis

**Affiliations:** 1 Division of Basic Neurosciences, Biomedical Research Foundation of the Academy of Athens, Athens, Greece; 2 Neuroscience Lab, University of Ottawa, Ottawa, Ontario, Canada; 3 Second Department of Neurology, University of Athens Medical School, Athens, Greece; University of Nebraska, United States of America

## Abstract

**Background:**

The mechanisms through which aberrant α-synuclein (ASYN) leads to neuronal death in Parkinson's disease (PD) are uncertain. In isolated liver lysosomes, mutant ASYNs impair Chaperone Mediated Autophagy (CMA), a targeted lysosomal degradation pathway; however, whether this occurs in a cellular context, and whether it mediates ASYN toxicity, is unknown. We have investigated presently the effects of WT or mutant ASYN on the lysosomal pathways of CMA and macroautophagy in neuronal cells and assessed their impact on ASYN-mediated toxicity.

**Methods and Findings:**

Novel inducible SH-SY5Y and PC12 cell lines expressing human WT and A53T ASYN, as well as two mutant forms that lack the CMA-targeting motif were generated. Such forms were also expressed in primary cortical neurons, using adenoviral transduction. In each case, effects on long-lived protein degradation, LC3 II levels (as a macroautophagy index), and cell death and survival were assessed. In both PC12 and SH-SY5Y cycling cells, induction of A53T ASYN evoked a significant decrease in lysosomal degradation, largely due to CMA impairment. In neuronally differentiated SH-SH5Y cells, both WT and A53T ASYN induction resulted in gradual toxicity, which was partly dependent on CMA impairment and compensatory macroautophagy induction. In primary neurons both WT and A53T ASYN were toxic, but only in the case of A53T ASYN did CMA dysfunction and compensatory macroautophagy induction occur and participate in death.

**Conclusions:**

Expression of mutant A53T, and, in some cases, WT ASYN in neuronal cells leads to CMA dysfunction, and this in turn leads to compensatory induction of macroautophagy. Inhibition of these lysosomal effects mitigates ASYN toxicity. Therefore, CMA dysfunction mediates aberrant ASYN toxicity, and may be a target for therapeutic intervention in PD and related disorders. Furthermore, macroautophagy induction in the context of ASYN over-expression, in contrast to other settings, appears to be a detrimental response, leading to neuronal death.

## Introduction

α-Synuclein (ASYN) is an abundant neuronal protein closely linked to Parkinson's Disease (PD) pathogenesis [1]–[3]. Missense mutations in the gene encoding ASYN [4]–[6] and multiplications of the ASYN gene locus lead to familial cases of PD [7]–[9]. Even sporadic PD cases are genetically linked to ASYN polymorphisms, which may modulate ASYN transcription [10]. Furthermore, ASYN is widely considered to be the main element of Lewy Bodies (LBs) that characterize PD pathologically [11]. ASYN deposition occurs early in PD, before overt motor symptoms [12]. Aging in humans and monkeys is associated with an increase of ASYN protein levels in the substantia nigra, and this increase correlates with dopaminergic dysfunction [13]. Cellular or animal models based on overexpression of ASYN demonstrate neuronal dysfunction and, occasionally, death, as well as inclusion formation and motor phenotypes [14]. These data, in conjunction, have led to the idea that ASYN is linked to PD through a toxic gain of function that is latent in the WT protein, and is manifest when levels of WT ASYN increase, when point mutations occur, or when WT ASYN is post-translationally altered, through oxidation, nitration or other modifications [15].

Various theories have been proposed to explain this toxic gain of function of ASYN. The feature that has attracted the most attention is the propensity of ASYN to misfold, assume beta-sheet structures, and fibrillize first into intermediate soluble 〈〈protofibrillar〉〉 or 〈〈oligomeric〉〉 species and then to mature fibrils. Something along this aggregation pathway is posited to be toxic to neuronal cells. The weight of the evidence favours the idea that the intermediate oligomeric species are the main culprits [14]. Such species could form pores on membranes of cells or intracellular organelles, such as mitochondria or vesicles, or aberrantly interact with cellular proteins, or, due to their abnormal structure, disrupt normal cellular processes, such as ER-Golgi trafficking or proteasomal degradation [14], [16]–[20].

In prior work, we had noted that expression of mutant A53T ASYN in PC12 cells led to accumulation of autophagic vacuoles and lack of lysosomal acidification. This had led us to propose that lysosomal alterations may be a direct consequence of aberrant ASYN expression [18]. A possible mechanism for this effect emerged when we demonstrated in *in vitro* assays, using purified ASYN protein and isolated liver lysosomes, that mutant A30P and A53T ASYNs inhibit uptake and degradation of Chaperone-Mediated Autophagy (CMA) substrates [21]. CMA is one of 3 major pathways of lysosomal degradation of intracellular proteins. The other two are microautophagy and macroautophagy [22], [23]. CMA involves the selective targeting of proteins containing a KFERQ peptide motif to lysosomes. This requires binding to the lysosomal receptor, Lamp2a, the rate-limiting step in CMA [24]–[26]. In a more recent study, we have shown that WT ASYN is degraded through CMA in human SH-SY5Y, rat PC12 cells and primary neurons [27].

Despite the demonstration of CMA blockade by mutant ASYNs in the *in vitro* assay with purified proteins and isolated liver lysosomes, it remains unclear whether such blockade can be conferred in a cellular context, and whether it could be responsible for the lysosomal dysfunction that has been observed in certain instances when ASYN is overexpressed. Furthermore, it is unclear to what extent CMA blockade and resultant lysosomal alterations could be related to the toxicity conferred by aberrant ASYN.

A related question concerns the role of macroautophagy in ASYN-induced death. Reports in various cell systems and mice have stressed the importance of macroautophagy for cell survival in various neurodegenerative diseases and suggest induction of macroautophagy as a therapeutic strategy, while other studies suggest that suppression of macroautophagy is protective [28]–[35]. Furthermore, given that macroautophagy is also involved in ASYN clearance [27], [36], its enhancement has been proposed as therapeutic strategy in PD.

In the current work, we have attempted to address these issues, taking advantage of the creation of artificial mutants of ASYN that are not targeted to the CMA pathway, and thus would not be expected to compromise it. Apart from the role of CMA in ASYN-mediated toxicity, we have also evaluated the role of macroautophagy in this process.

## Results

### CMA dysfunction is responsible for the decrease of total lysosomal protein degradation in rat PC12 cell lines inducibly expressing human A53T ASYN

**Figure 1:**
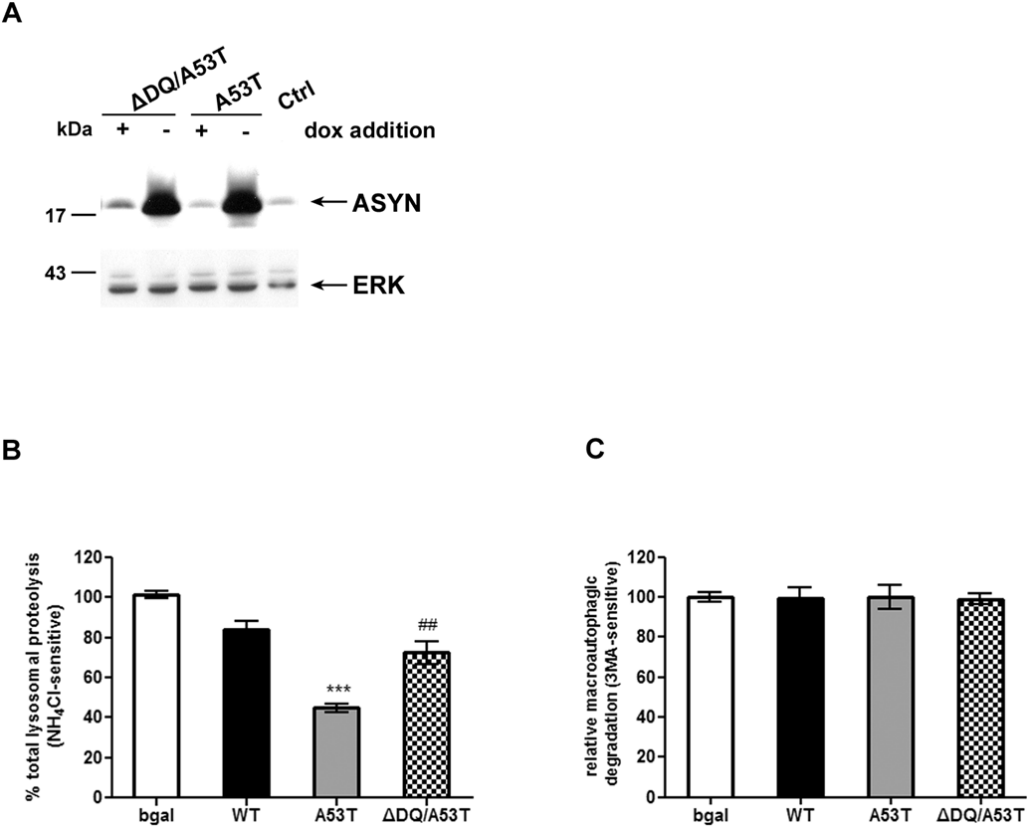
Over-expression of A53T ASYN in PC12 cells results in lysosomal dysfunction due to CMA impairment. (A) Generation of stable inducible Tet-Off PC12 cell lines over-expressing human A53T and ΔDQ/A53T ASYN. Cells were cultured in the presence (+) or absence (−) of dox (2 µg/ml) for 4 days and assayed for ASYN expression with the C20 polyclonal Ab. ERK Ab is used as a loading control. (B, C) PC12 cells stably transfected with WT or mutant ASYNs (A53T, ΔDQ/A53T) or control bgal were labeled with [^3^H] leucine for 48 hrs (2 µCi/ml). Cells were treated with or without NH_4_Cl (25 mM) or 3MA (10 mM) and degraded proteins were assayed 14 hrs later. Rate of total (B) (inhibitable by NH_4_Cl) and (C) of macroautophagic (inhibitable by 3MA) long lived protein degradation in PC12 cell lines expressing bgal, WT, A53T or ΔDQ/A53T ASYN. For each line, protein degradation was assessed in the presence or absence of dox, and the results are reported as percentage degradation in the absence compared to the presence of dox (induced vs. non-induced). All presented data are the mean of 3 independent experiments and within each experiment triplicate samples per condition were assessed. (***p<0.001, one way ANOVA followed by the Student-Newman-Keuls' test, comparing between cells expressing all forms of ASYN and bgal controls; ^##^ *p*<0.01, comparing between cells expressing A53T and ΔDQ/A53T ASYN).

We have previously shown that purified mutant ASYN inhibits the *in vitro* uptake of CMA substrates in isolated lysosomes [21]. Furthermore, mutant ASYN expression impairs lysosomal acidification and lysosomal-dependent protein degradation in the PC12 cell model [18], [21]. It has not been proven however that mutant ASYN can induce CMA impairment in a cellular context, or that this effect could lead to lysosomal dysfunction. To test this hypothesis, we generated stable inducible Tet–off PC12 cell lines overexpressing human A53T ASYN and lines expressing ΔDQ/A53T ASYN. The ΔDQ mutant lacks the CMA targeting motif, and was previously shown to lack CMA–dependent uptake into lysosomes [21]; therefore, the double mutant ΔDQ/A53T should not be targeted to the CMA pathway and, despite the presence of the A53T mutation, should not inhibit it. The prediction, if the hypothesis holds true, would be that, whatever the lysosomal effects induced by A53T, they would not occur when ΔDQ/A53T was expressed. We also used cells inducibly expressing WT ASYN or bgal, which have been previously described [27]. Inducible expression was found to be similar between cell lines (Figure 1A). In order to measure and compare lysosomal function in these cell lines, we employed the long lived protein degradation assay using specific pharmacological inhibitors to discriminate between the different forms of lysosomal degradation. In this manner we found that cells expressing the A53T mutant form of ASYN exhibited reduced total lysosomal protein degradation (inhibited by NH_4_Cl) by ∼30%. In contrast, the ΔDQ/A53T ASYN-expressing cells showed no change (Figure 1B) and were at similar levels with those in WT ASYN and control bgal expressing cells (Figure 1B). The observed reduction in total lysosomal degradation could be attributed to a possible reduction in macroautophagy or CMA, as microautophagy is not thought to play a significant role in bulk lysosomal protein degradation. However, the levels of macroautophagic degradation (inhibited by 3MA) were not altered in any of the cell lines (Figure 1C). Additionally, there was no change in the conversion of the cytoplasmic form of LC3 (LC3-I, 18 kDa) to the preautophagosomal and autophagosomal membrane-bound form of LC3 (LC3-II, 16 kDa) by western blot, an index of mature autophagosomes (data not shown). Therefore, we conclude that the reduction in total lysosomal degradation in the A53T ASYN expressing lines is due to a reduction in CMA-dependent degradation and to the presence of the CMA targeting motif.

We wished to examine whether the observed CMA-lysosomal dysfunction induced by A53T ASYN expression could lead to cellular toxicity. Induction of ASYN for at least 10 days in mitotic PC12 cells had no obvious effect on cell survival, as judged by Hoechst and Ethidium Homodimer co-staining (Supplementary Figure S1A). Therefore, although induction of A53T ASYN causes lysosomal impairment in cycling PC12 lines, this does not result in increased cell death in this particular context.

### CMA dysfunction is responsible for the decrease of total lysosomal protein degradation in human SH-SY5Y cell lines inducibly expressing human A53T ASYN

**Figure 2:**
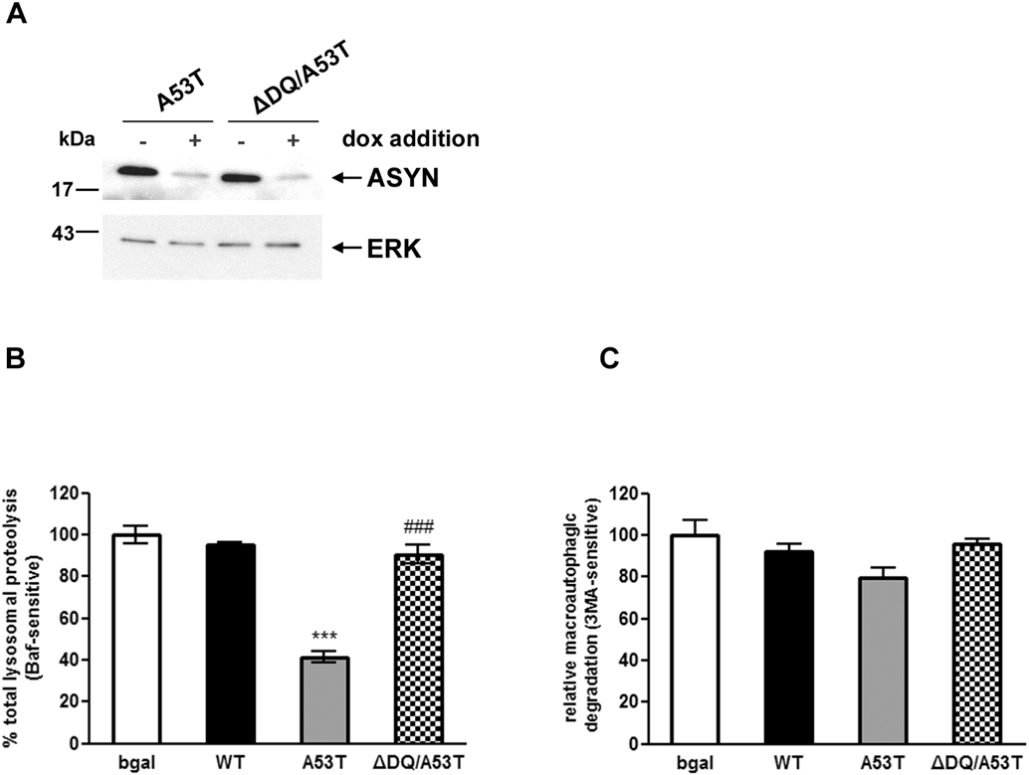
Over-expression of A53T ASYN impairs lysosomal function due to CMA targeting in proliferating SH-SY5Y cells. (A) Generation of stable inducible Tet-Off SH-SY5Y cell lines over-expressing human A53T and ΔDQ/A53T ASYN. The cells were cultured in the presence (+) or absence (−) of dox (3 µg/ml) for 4 days and assayed for ASYN expression with the C20 polyclonal Ab. ERK Ab is used as a loading control. (B, C) SH-SY5Y cells stably transfected with WT or mutant ASYNs (A53T, ΔDQ/A53T) or control bgal were labeled with [^3^H] leucine for 48 hrs (2 µCi/ml). Cells were treated with or without Baf (500 nM) or 3MA (10 mM) in serum free medium and degraded proteins were assayed 14 hrs later. Rate of total (B) (inhibitable by Baf) and (C) of macroautophagic (inhibitable by 3MA) long lived protein degradation in SH-SY5Y cells expressing bgal, WT, A53T or ΔDQ/A53T ASYN. For each line, protein degradation was assessed in the presence or absence of dox, and the results are reported as percentage degradation in the absence compared to the presence of dox (induced vs. non-induced). All presented data are the mean of 3 independent experiments and within each experiment triplicate samples per condition were assessed. (***p<0.001, one way ANOVA followed by the Student-Newman-Keuls' test, comparing between cells expressing all forms of ASYN and bgal controls; ^###^ *p*<0.001, comparing between cells expressing A53T and ΔDQ/A53T ASYN).

We went on to examine whether the A53T mutation has similar effects on lysosomal protein degradation in human SH-SY5Y cell lines. We generated stable inducible Tet–off SH-SY5Y cell lines over-expressing human A53T ASYN or the ΔDQ/A53T ASYN that lacks the CMA recognition motif. WT ASYN and bgal expressing cells, reported previously [27], were also used. Inducible expression was found to be similar between cell lines (Figure 2A). We detected a significant reduction in the levels of total lysosomal protein degradation (inhibited by Bafilomycin) by ∼40% (+/−) in the A53T ASYN expressing cells (Figure 2B). In contrast, ΔDQ/A53T ASYN expressing cells had similar levels of total lysosomal protein degradation to the levels observed for WT or bgal-expressing cells (Figure 2B). We observed no significant changes in the levels of macroautophagic protein degradation (inhibited by 3MA) (Figure 2C) or an increase in the levels of LC3-II (data not shown). We therefore concluded that, similar to our result with PC12 cells, the reduction in total lysosomal protein degradation in A53T ASYN expressing human cell lines was also due to CMA impairment. These lysosomal effects did not however affect neuronal survival in these cycling cells (Supplementary Figure S1B).

### CMA impairment mediates lysosomal dysfunction in neuronally differentiated SH-SY5Y cells expressing human WT ASYN

**Figure 3:**
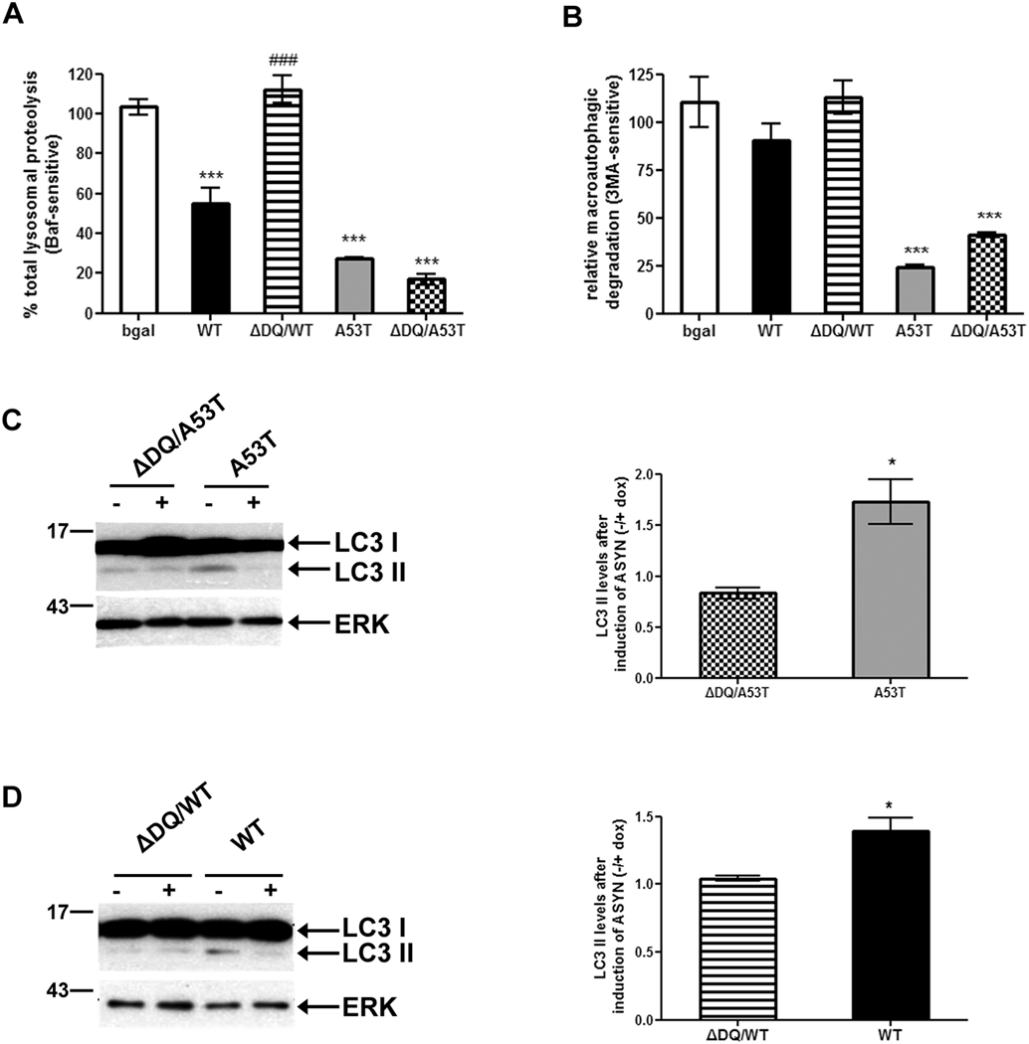
Over-expression of ASYNs impairs lysosomal function in differentiated SH-SY5Y cells, dependent on CMA targeting. (A, B) SH-SY5Y cells expressing WT or mutant ASYNs were differentiated in 20 µM RA for 5 days in the presence or absence of dox and then treated as in Figure 2. Rate of total (A) and (B) macroautophagy-dependent long lived protein degradation in differentiated SH-SY5Y cells expressing ASYN (WT, ΔDQ/WT, A53T or ΔDQ/A53T). Bgal expressing cells are used as controls. For each line, protein degradation was assessed in the presence or absence of dox, and the results are reported as percentage degradation in the absence compared to the presence of dox (induced/non-induced). All presented data are the mean of 4 independent experiments and within each experiment triplicate samples per condition were assessed. (***p<0.001, one way ANOVA followed by the Student-Newman-Keuls' test, comparing between cells expressing all forms of ASYN and control bgal; ^###^ *p*<0.001, comparing between cells expressing WT and ΔDQ/WT ASYN). (C, D) Western blotting analysis of differentiated SH-SY5Y cells (7 days, +/−dox) expressing A53T, ΔDQ/A53T, WT or ΔDQ/WT ASYN. ERK Ab is used as a loading control. Representative immunoblots of LC3 II are presented in the left panels and quantification of LC3 II levels after ASYN induction (−dox compared to +dox) is shown in the right panels. All results are expressed as the ratio of OD values to the corresponding controls and data are presented as mean of ±S.E. of 3 independent experiments [*p<0.05, Student's t-test comparing between cells expressing A53T and ΔDQ/A53T (C), or between cells expressing WT and ΔDQ/WT ASYN (D)].

We have previously found that upon neuronal differentiation SH-SY5Y cells become vulnerable to WT ASYN-mediated toxicity (Vekrellis et al., in press). We wished therefore to examine the status of the lysosomal degradation system in this setting. Accordingly, SH-SY5Y cells were differentiated with all trans Retinoic Acid (RA) for up to 5 days along with the simultaneous induction of various forms of ASYN. In differentiated SH-SY5Y cells expressing A53T ASYN we observed a substantial inhibition of total lysosomal protein degradation, more dramatic than the one detected in their cycling counterparts. In contrast to the cycling cell model, macroautophagic protein degradation was also severely compromised (Figure 3A, B). ΔDQ/A53T expression led to a similar degree of lysosomal dysfunction as A53T. Therefore, in this neuronally differentiated cellular setting, A53T seemed to cause lysosomal damage that was more generalized and not confined to CMA. Interestingly, despite the decrease of macroautophagy-dependent degradation, there was a significant increase of the levels of LC3-II in A53T ASYN-expressing cells (Figure 3C), suggesting that macroautophagosomes were accumulating. ΔDQ/A53T ASYN expression, in contrast, did not lead to LC3-II increase (Figure 3C).

In differentiated cells overexpressing WT ASYN, and in contrast to their cycling counterparts, we detected a decrease in the rate of total lysosomal protein degradation by ∼40% that was not observed in cells expressing ΔDQ/WT ASYN (Figure 3A). Induction of WT ASYN resulted in increased levels of LC3-II (Figure 3D), but without a significant increase of macroautophagic protein degradation (inhibited by 3MA) (Figure 3B). These phenomena were not observed in ΔDQ/WT ASYN-expressing cells, indicating that in neuronally differentiated SH-SY5Y cells, WT ASYN causes lysosomal alterations (general lysosomal dysfunction and LC3-II induction) mainly due to CMA impairment.

### Toxicity induced by over-expression of WT and mutant ASYNs in differentiated SH-SH5Y cells correlates with CMA impairment and induction of macroautophagy

**Figure 4:**
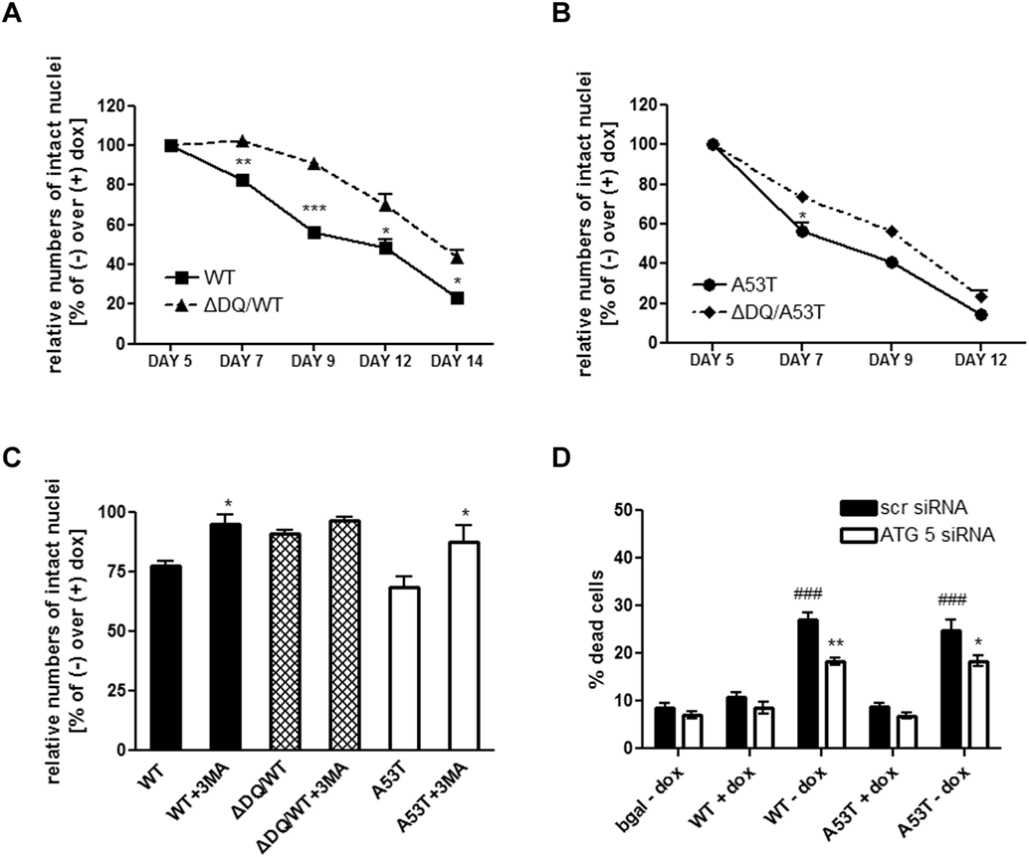
ASYN-induction is toxic to differentiated SH-SH5Y cells due to CMA blockade and macroautophagy induction. (A, B) ASYN (WT, ΔDQ/WT, A53T or ΔDQ/A53T)-expressing SH-SH5Y cells, were differentiated in RA (+/−dox). Samples were collected at 5, 7, 9, 12, 14 days after RA addition and survival was assessed by counting the number of intact nuclei. Rate of survival, presented in each case as the percentage of the (−) over the (+) dox condition, is shown for WT/ΔDQ and WT ASYN (A), and for A53T/ΔDQ and A53T ASYN (B) cells. All presented data are the mean of 3 independent experiments. Within each experiment triplicate samples per condition were assessed. (*p<0.05, **p<0.01, ***p<0.001, Student's t-test comparing WT or A53T ASYN-expressing cells and their corresponding ΔDQ mutants). (C, D) Suppression of macroautophagy with 3MA (C) or with ATG 5 siRNA (D), rescues differentiated SH-SY5Y cells from ASYN-induced death. (C) ASYN (WT, ΔDQ/WT, A53T)-expressing SH-SY5Y cells, were differentiated (+/−dox) for 5 days before 3MA addition. 36 hrs later, survival was assessed as in A, B. (*p<0.05, student's t-test, comparing WT or A53T-expressing cells+/−3MA). (D) WT or A53T ASYN cells and bgal cells were differentiated for 5 days (+/−dox) and transfected with scrambled (scr) or ATG 5 siRNA together with EGFP. Cell death was assessed 72 hrs later by counting the percentage of EGFP-positive transfected cells that were also Ethidium Homodimer-positive. At least 100 EGFP-positive cells were counted per well per condition. The data are presented as mean±SE of 3 independent experiments. Within each experiment triplicate samples per condition were assessed (^###^ *p*<0.001, one way ANOVA followed by the Student-Newman-Keuls' test, comparing WT or A53T ASYN cells+/−dox; *p<0.05, **p<0.01, comparing WT or A53T ASYN-induced cells (−dox) transfected with ATG 5/EGFP to the control scr/EGFP transfected cells).

Our results in differentiated SH-SY5Y cells indicated that induction of WT or mutant ASYN causes lysosomal dysfunction and induction of autophagosome formation, as defined by LC3-II accumulation. We wished to examine whether these effects could be correlated with cell toxicity. In contrast to our observations in proliferating cells, WT or A53T ASYN expression in neuronally differentiated SH-SH5Y cells resulted in a gradual induction of cell death (Figure 4A, B). The death induced by WT ASYN in this context has been previously reported (Vekrellis et al., in press). The rate of death was significantly reduced in ΔDQ/WT-expressing compared to WT ASYN-expressing cells (Figure 4A), and was slightly attenuated (with significance at one time point) in ΔDQ/A53T ASYN-expressing compared to A53T ASYN-expressing cells. These results suggest that CMA impairment induced by WT ASYN expression is partly responsible for the death observed in differentiated SH-SY5Y cells. In the case of A53T, the contribution of CMA targeting to death was more marginal, but still present.

As neuronal differentiation of our cultures resulted in increased conversion of LC3-I to LC3-II following WT or A53T ASYN induction (Figure 3C, D), we wanted to investigate the role of macroautophagy in the observed cell death. We used the pharmacological macroautophagy inhibitor 3-methyladenine (3MA), or a small interfering RNA against the human autophagy gene ATG 5 to inhibit macroautophagy in our cutures. 3MA was added to the culture medium before death features started to appear. 36 hours after 3MA addition we observed an increase in the survival of differentiated WT ASYN expressing cells from 77.7±1.6% (WT–dox) to 95.2±4% (WT-dox+3MA) and similarly in A53T ASYN expressing cells from 68.8±4% (A53T–dox) to 87.8±6% (A53T-dox+3MA) (Figure 4C). The ΔDQ/WT-expressing cells did not show any differences in survival+/−3MA.

We have previously shown that inhibition of macroautophagy with 3MA, leads to WT ASYN accumulation in cycling SH-SY5Y cells [27] and we also observed an accumulation of WT and A53T ASYN in differentiated cells (Supplementary Figure S2). Therefore, 3MA exerts protective effects in this model, despite an increase of ASYN levels.

Since autophagy requires the expression of autophagy genes such as beclin-1, ATG 7 and ATG 5 to form autophagosomes [37], [38], we hypothesized that the suppression of ATG-5 expression will also decrease WT and A53T ASYN induced cell death. In differentiated WT and A53T ASYN overexpressing cells (8 days after RA addition), expression of ATG 5 was suppressed with small interfering RNA (siRNA) against ATG-5 (Supplementary Figure S3). Neuronal survival was determined 72 hrs later as described in Materials and Methods. ATG 5 siRNA significantly decreased the level of WT and A53T ASYN-induced cell death (Figure 4D). Taken together, these results indicate that human WT and A53T ASYN induce autophagic death in human differentiated SH-SY5Y cells, a death that depends on the activation of macroautophagy.

### Adenoviral mediated over-expression of human A53T ASYN in rat primary cortical neurons results in CMA dysfunction, followed by a compensatory increase in macroautophagy

**Figure 5:**
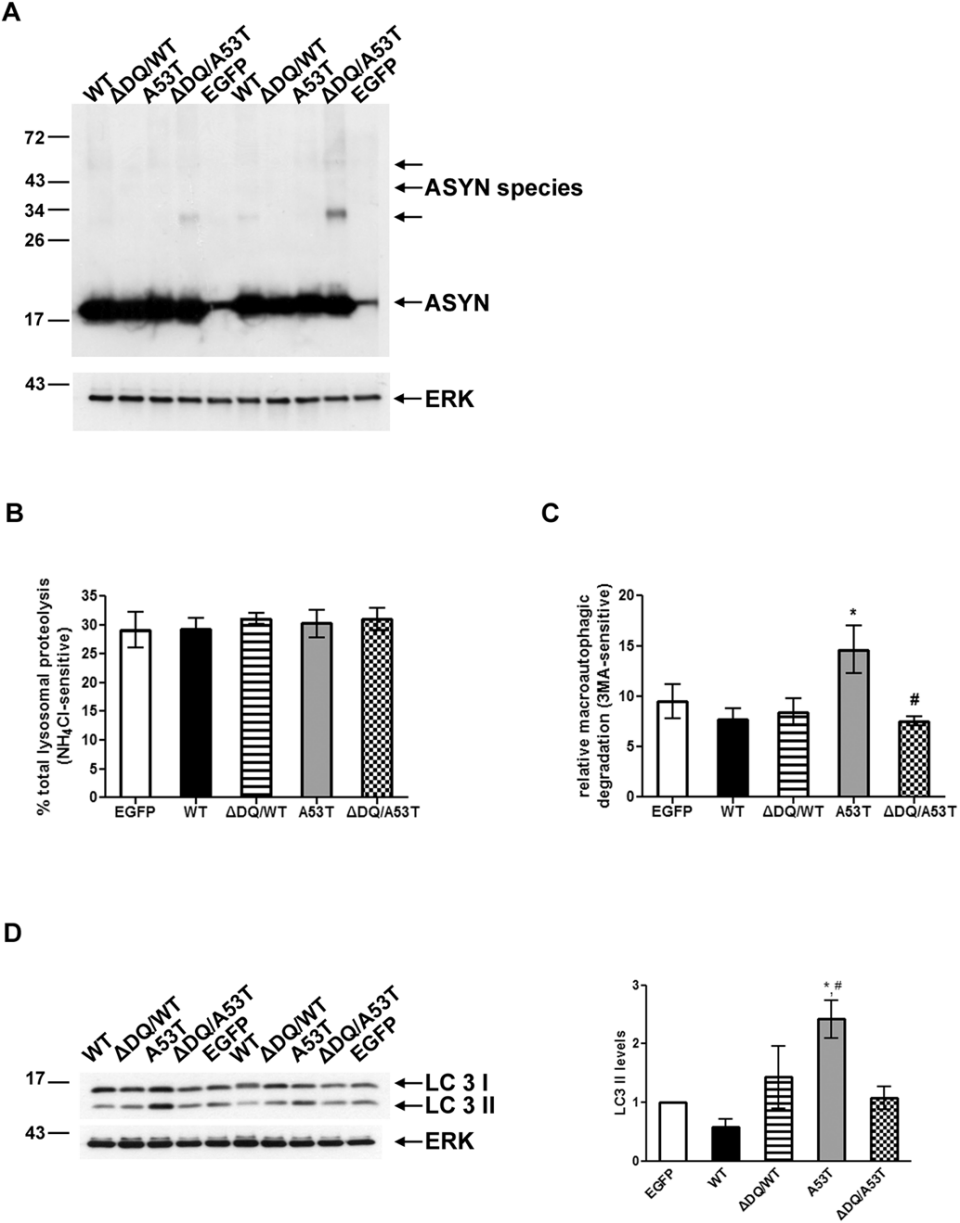
Over-expression of A53T ASYN in primary cortical neurons alters lysosomal function dependent on CMA targeting. (A) Five day-old cortical cultures were transduced with adenoviruses (MOI 150) expressing human ASYN (WT, ΔDQ/WT, A53T or ΔDQ/A53T) or EGFP (control virus) as described in Materials and Methods. ASYN expression was assessed 96 hrs later by performing immunoblotting with the C20 polyclonal Ab. ERK Ab is used as a loading control. A representative immunoblot of ASYN expression is shown. (B, C) Rate of total (inhibitable by NH_4_Cl) and of macroautophagic (inhibitable by 3MA) long lived protein degradation in rat cortical cultures, 96 hrs after transduction with adenoviruses expressing WT or mutant ASYNs. EGFP transduced neurons are used as controls. All presented data are the mean of 4 independent experiments and within each experiment triplicate samples per condition were assessed. (D) Cortical neurons were treated as in B, lysed and assessed by western immunoblotting for LC3 II levels. ERK Ab is used as a loading control. Representative immunoblot of LC3 II is presented in the left panel and quantification of LC3 II levels after WT or mutant ASYN transduction is shown in the right panel. All results are expressed as the ratio of OD values to the corresponding controls and all data are presented as mean of ±S.E. of 4 independent experiments (^*^ *p*<0.05, one way ANOVA followed by the Student-Newman-Keuls' test, comparing between cultures expressing various forms of ASYN and control EGFP; ^#^ *p*<0.05, comparing between cultures transduced with A53T and ΔDQ/A53T ASYN).

We sought to examine whether over-expression of human WT or mutant forms of ASYN impacts lysosomal function and contributes to neuronal toxicity in primary cortical rat cultures. These cultures provide a rich, homogeneous source of material of CNS neurons, in which biochemical studies can be performed. Cortical neurons are severely affected in synucleinopathies, especially Lewy Body Dementia (LBD) and PD with Dementia (PDD). For this purpose, we generated adenoviruses expressing WT and A53T ASYN, as well as the two mutant forms (ΔDQ/WT and ΔDQ/A53T) that lack the CMA-targeting motif and a control virus expressing the EGFP protein. We transduced five day-old cortical cultures with these viruses with multiplicities of infection (MOIs) ranging from 50–150 for 24 h and first tested the expression of human ASYN 24 to 96 h later by performing immunostaining and immunoblotting with a specific antibody against human ASYN (LB 509, data not shown). All forms of ASYN were expressed in equally high levels, as assessed with a polyclonal antibody that also recognizes endogenous ASYN (Figure 5A). At longer exposures higher molecular weight species were readily detectable in the lysates of ASYN transduced cortical cultures and absent in control EGFP transduced neurons (data not shown). We assessed the impact of the over-expression of various forms of ASYN on protein degradation, as above, at 4 days and 1 week following viral transduction. When compared to EGFP-transduced cultures, total long-lived protein degradation (inhibited by NH_4_Cl) was not affected with transduction of any of the ASYN constructs (Figure 5B). On the other hand, we observed a significant induction of macroautophagic degradation (3MA-dependent) in cultures expressing A53T ASYN, compared to those expressing EGFP (Figure 5C). Furthermore, A53T ASYN caused a significant reduction on CMA-dependent proteolysis (Supplementary Figure S4). These effects did not occur when ΔDQ/A53T ASYN was expressed, suggesting that the induction of macroautophagy-dependent degradation by A53T ASYN occurred secondary to CMA targeting. Macroautophagy induction by A53T ASYN was further confirmed by the conversion of LC3-I toLC3-II (Figure 5D), which did not occur with ΔDQ/A53T ASYN expression, consistent with the long-lived degradation results. The above results suggest that over-expression of human A53T ASYN in primary neurons causes significant reduction of CMA activity, which is followed by a compensatory increase in macroautophagy, dependent on CMA targeting.

### Over-expression of human ASYN is toxic to cortical neurons

**Figure 6:**
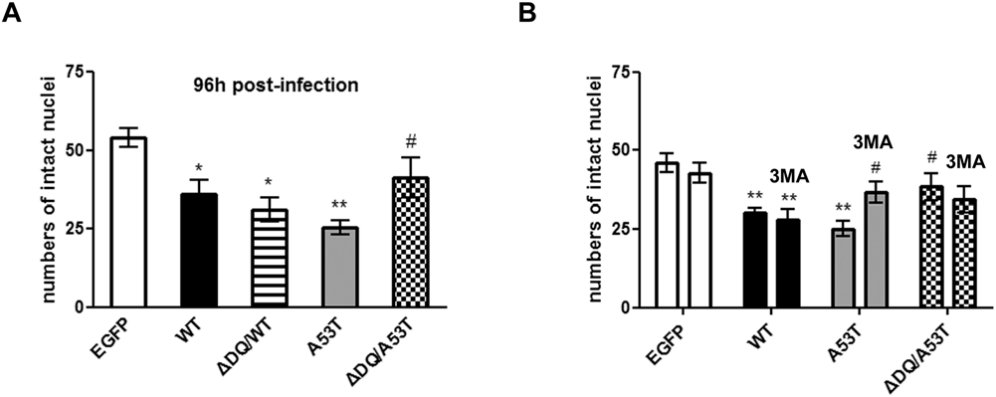
Over-expression of human ASYN (WT, mutants) is toxic to rat cortical neurons. (A) Five days-old cortical cultures were transduced with adenoviruses (MOI 150) expressing human ASYN (WT, ΔDQ/WT, A53T or ΔDQ/A53T) or EGFP (control virus) as described in Materials and Methods. 96 hrs later cells were lysed with a nuclear-sparing buffer and intact nuclei were counted in a hemacytometer. (B) Cortical cultures were treated as in A with slight modifications. 72 hrs post-infection, 3MA (10 mM) was added and intact nuclei were counted 24 hrs later. All data are presented as mean±SE of independent experiments and within each experiment triplicate samples per condition were assessed. (*p<0.05, **p<0.01, one way ANOVA followed by the Student-Newman-Keuls' test, comparing between cultures expressing various forms of ASYN and control EGFP; ^#^ *p*<0.05 comparing between cultures transduced with A53T and ΔDQ/A53T ASYN or between A53T ASYN transduced neurons+/−3MA).

Many studies have reported that over-expression of human WT or mutant ASYN in cultured primary neurons and animal models is associated with reduced cell viability [39]–[42]. The availability of the ΔDQ mutants enabled us to examine the issue of the relationship of CMA dysfunction to ASYN toxicity in primary neurons. We therefore investigated first whether the adenoviral over-expression of WT or mutant ASYNs affects the survival of rat cortical neurons. As shown in Figure 6A, over-expression of all forms of ASYN resulted in significant reduction of cell viability of cortical neurons (96 hours post-infection), compared to EGFP overexpression, with A53T ASYN exhibiting the most toxic effect. Interestingly, ΔDQ/A53T ASYN was significantly less toxic than A53T ASYN, suggesting that CMA targeting may mediate some of the aberrant effects of A53T ASYN. There was no significant survival difference between WT and ΔDQ/WT ASYN (Figure 6A).

As we had observed that A53T ASYN induces upregulation of macroautophagy secondary to CMA targeting, we wished to determine whether this induction of macroautophagy could be related to the observed death. We therefore investigated whether inhibition of macroautophagy could be protective. For this purpose, we treated cortical neurons with 3MA (10 mM, 24 h) following infection with EGFP, WT, A53T or ΔDQ/A53T ASYN adenoviruses. Inhibition of macroautophagy improved the survival of A53T ASYN-infected cortical cultures compared to A53T ASYN alone, whereas 3MA application had no effect on the death induced by the other ASYN forms (Figure 6B). Therefore, the induction of macroautophagy by A53T ASYN expression mediated in part cellular toxicity.

### siRNA down-regulation of the autophagy related gene 5 (ATG 5) rescues human A53T ASYN-induced toxicity in rat cortical neurons

**Figure 7:**
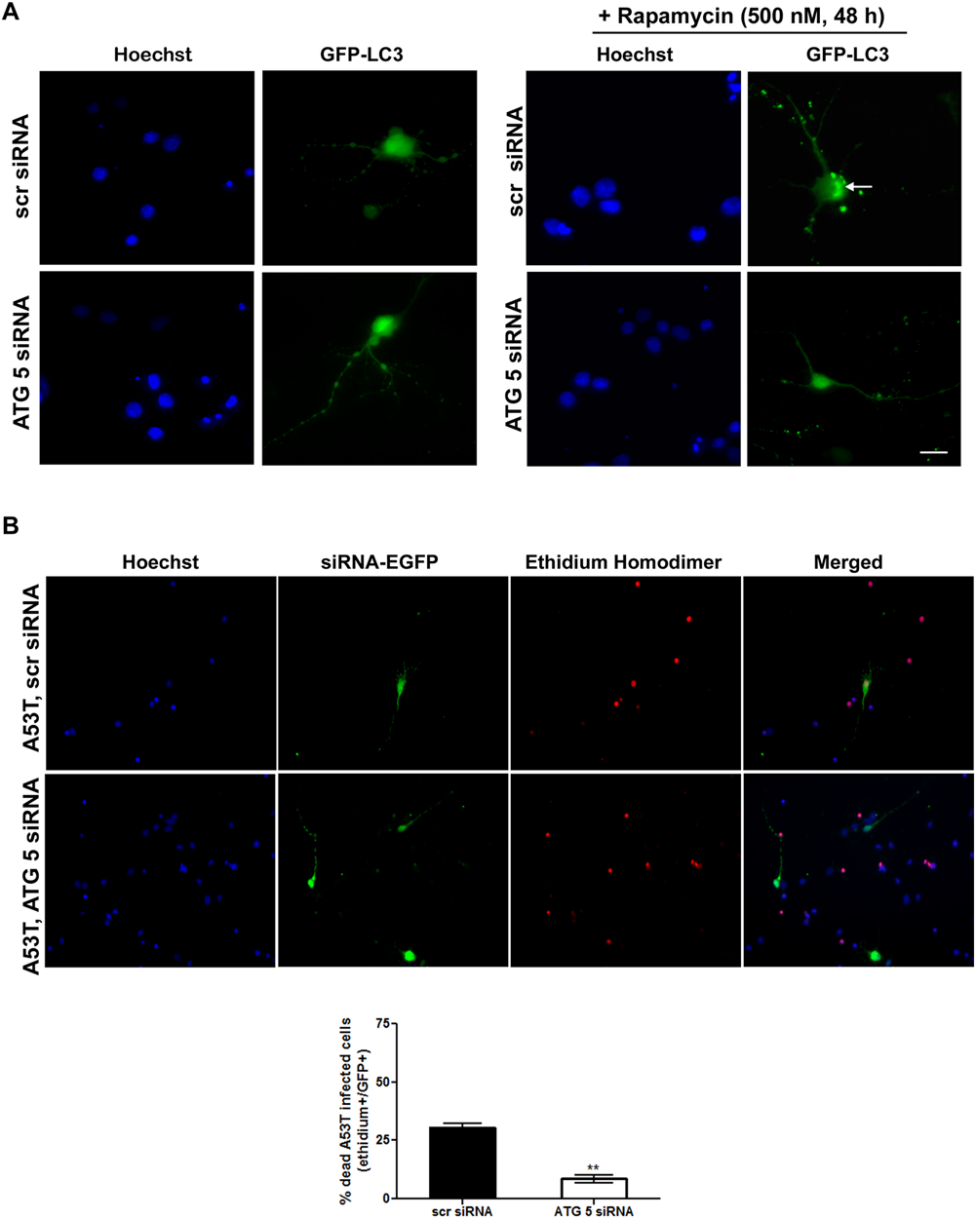
ATG 5 down-regulation decreases human A53T ASYN-induced death in rat cortical neurons. (A) Five day-old cortical cultures were transfected with GFP-LC3 cDNA together with the scrambled (scr) or the ATG 5 siRNA. 24 hrs later, rapamycin was added (500 nM, 48 hrs) and formation of GFP-LC3 vacuoles (dots) was determined by fluorescent microscopy. Representative fluorescent microscopic pictures showing the formation of GFP-LC3 vacuoles (indicated by the arrow) in cultures transfected with the GFP-LC3 construct together with the scr siRNA after rapamycin addition are shown. Vacuoles (dots) failed to be observed in rapamycin-treated cultures transfected with the GFP-LC3 construct together with the ATG 5 siRNA. (B) Five day-old cortical cultures were transduced with A53T ASYN adenovirus and 24 hrs later were transiently transfected with the scr or ATG siRNA along with an EGFP construct to monitor transfection. Cell death was assessed 72 hrs later by counting the percentage of EGFP-positive transfected cells that were positive for Ethidium Homodimer stain (dying cells). Representative pictures are shown in the upper panel and quantification of the percentage of scr or ATG5/EGFP positive cells that were also stained with Ethidium Homodimer is shown in the bottom panel. At least 100 EGFP-positive cells were counted per condition. The data are presented as mean±SE of 3 independent experiments (**p<0.01, Student's t-test, comparing A53T ASYN transduced neurons transfected with the scr or the ATG 5 siRNA. Bars: (A) 10 µM; (B) 50 µM.

From the aforementioned data it is evident that over-expression of human A53T ASYN induces cell death in primary cortical neurons, which can be attenuated by pharmacological suppression of macroautophagy. Following a similar approach with the one in differentiated SH-SY5Y neuroblastoma cells, and in order to confirm this finding using a molecular approach, we targeted the ATG 5 gene and examined whether the suppression of ATG 5 expression would also decrease A53T ASYN- induced cell death. To down-regulate ATG 5, we designed a siRNA (ATG 5) to the rat ATG 5 and a scrambled siRNA (scr), as a control. Because cortical neurons are post-mitotic cells, the classic method for siRNA delivery (with Lipofectamine) provides low transfection efficiency. We therefore initially examined whether we could sufficiently down-regulate ATG 5 in rat PC12 cells. When PC12 cells were transiently transfected with the ATG 5 siRNA, we detected a significant down-regulation of endogenous ATG 5 by almost 50% compared to cells transfected with the scr siRNA (Supplementary Figure S5). As expected, induction of autophagy with rapamycin (rap, 500 nM/24 h) increased LC 3-II levels in scr siRNA transfected PC12 cells (scr+rap: 2.5±0.4, compared to scr siRNA transfected untreated cultures 1.6±0.3), while, in the presence of rapamycin, siRNA down-regulation of ATG 5 abrogated the induction of LC3-II levels (ATG 5+rap: 1.4±0.3, compared to ATG 5 siRNA transfected untreated cultures 1.2±0.3) (Supplementary Figure S5). We then examined the effects of siRNA ATG 5 down-regulation in rat cortical cultures. We studied the impact of ATG 5 suppression on the formation of green fluorescent protein (GFP)-LC3 vacuoles (characteristic of autophagy). As shown in Figure 7A, ATG 5 siRNA down-regulation in the presence of rapamycin suppressed the formation GFP-LC3 vacuoles in cortical neurons (500 nM/48 h), while a punctate distribution of GFP-LC3 was apparent in scr siRNA-transfected cortical neurons treated with rapamycin, indicative of authophagic vacuole formation. Subsequently, we transduced 5 days-old cortical neurons with the A53T ASYN adenovirus and 24 h later we transiently transfected them with scr or ATG siRNA along with an EGFP vector to monitor transfection. ATG 5 down-regulation was associated with a significant reduction in neuronal death (8.5±1.5%, compared to 30.6±1.6% in scr siRNA transfected cells) (Figure 7B). Taken together, these data indicate that over-expression of A53T ASYN induces autophagic cell death in rat primary cortical cultures that is dependent upon macroautophagy induction, which in turn occurs due to CMA impairment.

### Dopamine-modified ASYN affects lysosomal function and survival of differentiated SH-SY5Y cells expressing WT ASYN

**Figure 8:**
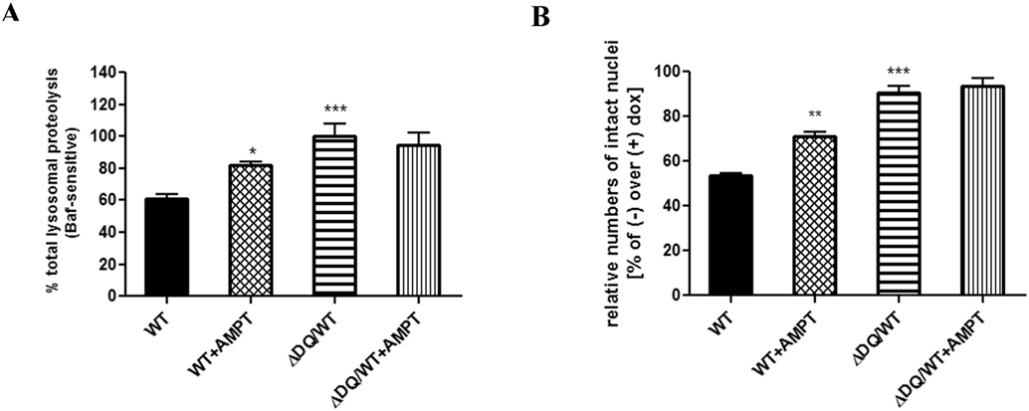
Reduction of dopamine levels improves lysosomal dysfunction and survival of differentiated SH-SY5Y WT ASYN expressing cells. (A, B) SH-SY5Y cells expressing WT or ΔDQ/WT ASYN were differentiated in RA (20 µM) in the presence (+) or absence (−) of dox (3 µg/ml) for 5 days before addition of alpha-mehtyl-p-tyrosine (AMPT, 1 mM). Next, cells were labeled with [^3^H] leucine for 48 hrs (2 µCi/ml) and degraded proteins were assayed 14 hrs later. (A) Rate of total long lived protein degradation in differentiated SH-SY5Y cell lines expressing WT ASYN+/−AMPT. ΔDQ/WT ASYN expressing cells are used as control. All data are presented as the relative percentage of long-lived protein degradation in the (−) relative to the (+) dox setting for each condition (B) Cells expressing WT or ΔDQ/WT ASYN were differentiated as in A for 5 days and AMPT was added for subsequent 3 days. Survival was assessed by counting the number of intact nuclei and is presented as (−) relative to (+) dox condition. All presented data are the mean of 3 independent experiments, and within each experiment triplicate samples per condition were assessed. (*p<0.05, **p<0.01, ***p<0.001, one-way ANOVA followed by the Student-Newman-Keuls' test, comparing between cells expressing WT ASYN+/−AMPT, and between cells expressing WT and ΔDQ/WT ASYN).

The differential effects of WT ASYN on CMA and the lysosomal system in the setting of neuronally differentiated SH-SY5Y cells, compared to the other cellular systems utilized, suggested to us that the dopaminergic phenotype of these cells may play a modifying role. Previous reports have shown that dopamine-modified WT ASYN, similar to the ASYN A53T and A30P mutants, inhibits CMA activity by blocking its own uptake and degradation, as well as the uptake and degradation of other CMA substrates [43]. This modified form of ASYN is suggested to be responsible for neuron toxicity and results from a non-covalent interaction of ASYN and oxidized dopamine [44]–[46]. To examine whether the aberrant effects of WT ASYN on lysosomal function and survival in differentiated SH-SY5Y cells were mediated by dopamine modification of ASYN, we used α-methyl-p-tyrosine (AMPT), an inhibitor of tyrosine hydroxylase (TH), the enzyme which is the rate-limiting step for dopamine production, and analysed long lived protein degradation and cell death in cells expressing WT ASYN. ΔDQ/WT ASYN-expressing cells were used as control. Pharmacological inhibition of TH by AMPT led to a significant recovery of lysosomal protein degradation (Fig. 8A), as well as to an improvement on the survival of WT ASYN-expressing cells (Fig. 8B). No such effects were seen in ΔDQ/WT ASYN-expressing cells. Taken together, these data suggest that dopamine modification of WT ASYN may in part be responsible for the reduction in CMA function and increased toxicity.

## Discussion

In the current study we have investigated the impact of WT and mutant forms of ASYN on lysosomal pathways in neuronal cells, as a possible pathogenetic mechanism for their toxic effects. For this purpose we have generated inducible rat PC12 and human SH-SY5Y cell lines expressing human WT and A53T ASYN, as well as two mutant forms (ΔDQ/WT and ΔDQ/A53T) that lack the CMA-targeting motif, and thus are not targeted to this pathway, do not interact with Lamp2a, and do not interfere with the degradation of other CMA substrates. We have used a similar approach in cortical neurons, where we have overexpressed these ASYN forms using adenoviral transduction.

In proliferating PC12 and SH-SY5Y cells, expression of A53T ASYN caused CMA impairment, as indicated by the marked decrease of total lysosomal degradation, in the face of unaltered macroautophagic, i.e. 3-MA-dependent, degradation. The fact that CMA impairment is responsible for lysosomal dysfunction in this setting was confirmed by the lack of changes in lysosomal degradation in cells expressing the double mutant ΔDQ/A53T. These data show for the first time in a cellular context that targeting of A53T ASYN to CMA is responsible for the decrease of total lysosomal degradation in neuronal cells. Interestingly, in these cycling cells, such lysosomal dysfunction was not associated with cell death or with compensatory induction of macroautophagy, as indicated also by the lack of changes in the ratio of LC3-I to LC3-II.

The situation in cortical neuron cultures was somewhat different, but still some essential features of the effects of A53T ASYN on lysosomal pathways were confirmed in this primary neuron setting. A53T ASYN again caused impairment of CMA, although on this occasion this did not lead to global lysosomal dysfunction, due to the compensatory activation of macroautophagy, which was identified through two different assays, the induction of 3MA-dependent degradation and the increase of the LC3-II to LC3-I ratio. In this case therefore, the activation of macroautophagy and the accumulation of autophagosomes were “productive”, in that they led to degradation of substrate proteins within lysosomes. A53T ASYN caused death that was in excess of that conferred by WT ASYN, and this death, as well as the macroautophagy induction, was abrogated with the double mutant ΔDQ/A53T. These data raised the possibility that the compensatory induction of productive macroautophagy may have deleterious consequences. This hypothesis was confirmed through pharmacological and molecular inhibition of this process, which led to improved survival. Taken together, these data support the concept that A53T ASYN causes toxicity in primary cortical neurons in part through CMA dysfunction and resultant aberrant macroautophagy activation. Thus, they confirm in a neuronal cell context our original hypothesis that mutant ASYNs may confer toxicity via CMA blockade [21], and for the first time provide conclusive evidence that the compensatory activation of macroautophagy mediates death in this setting.

In neuronally differentiated SH-SY5Y cells, WT ASYN expression overall led to similar effects with the expression of A53T in cortical neuron cultures. As we have previously reported (Vekrellis et al., in press), cell death occurred in this setting, unlike the situation in the cycling cells. Death was associated with CMA inhibition and increase of autophagosome formation, as assessed by the conversion of LC3-I to LC3-II. However, in this case, this was not associated with an increase of macroautophagy-dependent degradation, and therefore represented “non-productive” macroautophagy. Lysosomal changes did not occur with expression of the ΔDQ mutant, and death was significantly attenuated, confirming that CMA inhibition played a role in these effects. As in cortical neuron cultures with A53T ASYN expression, survival was increased with pharmacological or molecular inhibitors of macroautophagy. Therefore, even non-productive macroautophagy can lead to neuronal death.

These data in neuronally differentiated SH-SY5Y cells are important, because they indicate that, in certain settings, WT ASYN can also act as a CMA blocker and this effect can lead to cell death. This has also been suggested by Yang et al. [47] and may relate to the fact that SH-SY5Y cells are dopaminergic, and dopamine may form adducts with WT ASYN that can act as CMA blockers in *in vitro* assays [43]. Consistent with this idea, our results suggest that inhibition of dopamine synthesis in this cell system leads to restoration of lysosomal function and improved survival (Fig. 8). This is significant, given that in the vast majority of PD patients it is the WT protein that is implicated as the pathogenetic agent.

Expression of A53T ASYN in neuronally differentiated SH-SY5Y cells led to more profound lysosomal dysfunction and accelerated death compared to WT ASYN expression. As with the WT protein, LC3-II accumulated, and this accumulation did not occur when the double mutant ΔDQ/A53T was expressed. However, A53T ASYN expression led also to profound inhibition of macroautophagy-dependent degradation, and this effect was not attenuated when ΔDQ/A53T was expressed. ΔDQ/A53T expression was marginally less toxic than A53T ASYN. These results suggest that A53T in this setting may also affect lysosomal function independent of CMA, through yet unknown mechanisms.

It is worth noting that WT and mutant ASYNs are toxic to differentiated but not proliferating neuroblastoma cells. The factors accounting for this difference could include subtle differences in the generated ASYN species, differences in clearance mechanisms, or involvement of cell cycle molecules or other proteins differentially expressed in the two states. It is possible that the lysosome plays a greater role in the degradation of proteins in the differentiated compared to the proliferating state. In support of this, total long lived lysosomal degradation (inhibited by Baf) in the cycling cells was detectable only after removal of serum from the medium (data not shown), suggesting that in their normal state these cells rely very little on lysosomes for protein turnover. Furthermore, the degree of lysosomal dysfunction caused by both WT and A53T ASYN is more prominent in the differentiated cells, and it is only in these cells that we detected LC3-II accumulation. Therefore, specific effects on lysosomes may mediate the preferential toxicity of aberrant ASYN in the neuronally differentiated state.

From our results, it appears that, depending on the exact context, CMA inhibition conferred by aberrant ASYN may, or may not, lead to induction of the process of macroautophagy. Massey et al. [48] were the first to report that specific CMA inhibition may lead to activation of macroautophagy, and we have also confirmed it [27]. In our current experiments, this effect, as assessed by LC3-II accumulation, occurred in cortical neurons and in differentiated SH-SY5Y cells, but not cycling cells. The reasons for these differences and the mechanisms through which such compensatory activation of macroautophagy occurs are unclear. It is worth noting however that in every case in which we observe such compensatory activation of macroautophagy, defined again as an increase of LC3-II to –I ratio, there is cell death, and this death is attenuated by macroautophagy inhibition.

**Figure 9:**
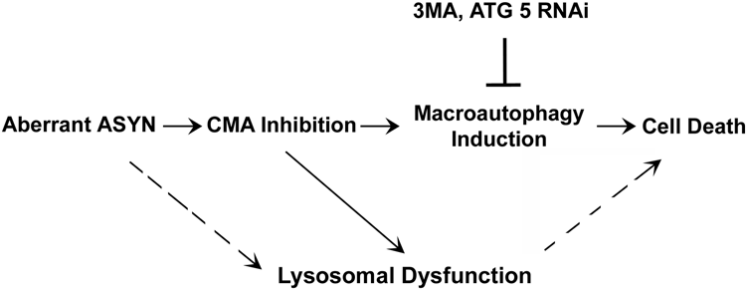
Schematic diagram of the lysosomal effects and relevant cell death pathways induced by aberrant ASYN. Aberrant ASYN induces in all settings CMA dysfunction. When this leads to compensatory macroautophagy induction (as assessed by autophagosome formation/accumulation), autophagic cell death, inhibited by macroautophagy inhibitors, occurs. In certain settings (A53T ASYN in neuronally differentiated SH-SY5Y cells), lysosomal dysfunction occurs independent of CMA targeting and may contribute to death (dotted arrows).

This raises the issue of the exact nature of the lysosomal effects of aberrant ASYN that are linked to toxicity. In contrast to macroautophagy induction which, as mentioned above, correlates with toxicity, lysosomal dysfunction (as defined by impairment of lysosomal-dependent long-lived protein degradation) does not. For example, no generalized lysosomal dysfunction occurs in cortical neurons, and yet there is lysosome-dependent death, and the converse is true in cycling cells. However, in cases where profound generalized lsysomal dysfunction occurs (the case of A53T ASYN expression in differentiated SH-SY5Y cells), this does appear to influence cell viability. It would appear therefore that autophagosome production/formation, and not general lysosomal dysfunction, is mainly responsible for the toxicity observed following CMA inhibition by aberrant ASYN (Fig. 9). As mentioned, autophagosome formation can exert toxicity regardless whether it leads to “productive” macroautophagy or not. Therefore, the toxic event appears to be the formation and accumulation of autophagosomes, and not their fusion with lysosomes or the excess degradation of substrate proteins. Such toxic effects could be related to progressive damage and destabilization of the membranes of the accumulating autophagic vacuoles, leading to the cytoplasmic release of vacuolar hydrolases and cell death [49], [50], or to impaired vesicular transport [51].

The role of macroautophagy in cell homeostasis remains controversial since macroautophagy contributes to cell survival under stress such as starvation, but can also contribute to cell death [37]. In particular as regards to neurodegeneration, this can apparently be induced both by lack and by excess of macroautophagy [52], [53] Regarding alpha-synucleinopathies, data, including our own [27], [36] and data presented here in supplementary Figure S2, suggest that various forms of ASYN may be degraded by this process, and that, therefore, a strategy of macroautophagy induction may be beneficial in terms of removing such aberrant species and thus preventing their toxic effects [29]. The data presented here though would argue that this represents an especially risky strategy, as, once ASYN levels accumulate and begin to exert toxic effects, they may do so in part via macroautophagy activation, and therefore further pharmacological macroautophagy activation may potentiate these effects.

It is worth noting in this context that toxicity induced by another genetic aberration leading to PD, that of mutant leucine rich repeat kinase 2 (LRRK2), has also been linked to macroautophagy induction. Inhibition of macroautophagy in differentiated SH-SY5Y cells reversed the detrimental effects of mutant G2019S LRRK2 on neuronal process length [54]. Decrease on neuronal process length represents a prominent feature of the degenerative phenotype associated with PD-associated LRRK2 mutations. Therefore, activation of macroautophagy may represent a more general mechanism through which aberrant forms of proteins linked to PD cause neurodegeneration.

We do not wish to imply that the lysosomal alterations presented here represent the only mechanisms through which ASYN exerts its toxicity. It is clear that this is part of the picture and that other processes play a role. Interestingly, monomeric ASYN is sufficient to exert CMA blockade [21], [43], and therefore the effects observed here cannot be accounted for by “fibrils” or “oligomers”. Other cellular effects mediated by such species may also be important for ASYN toxicity, as we and others have shown [55] (Vekrellis et al., in press). Despite this, it is obvious that targeting the CMA pathway may provide some therapeutic benefits in PD. Improving CMA function may not only serve to accelerate ASYN degradation, but also to mitigate potential deleterious consequences of aberrant ASYN on this system.

## Materials and Methods

### Generation of stable cell lines and transfections

Stable rat PC12 (Clontech) and human SH-SY5Y (gift from Dr. Darryl Yamashiro, Dept. of Pediatrics, Columbia University, USA) cells inducibly expressing human WT and ΔDQ/WT ASYNs, were generated as previously described [27]. Cells expressing human A53T and ΔDQ/A53T ASYNs were generated with similar procedures.

### Cell culture

PC12 cells were cultured in RPMI 1640 (Invitrogen) with 10% Horse Serum (Biowest) and 5% Fetal Bovine Serum (FBS, Biowest_._) on collagen coated plates. SH-SY5Y cells were cultured in RPMI 1640, 10% FBS. Stable cell lines were cultured with 200 µg/ml G418 and 25 µg/ml (PC12 cells) or 50 µg/ml (SH-SY5Y cells) Hygromycin B (Roche). Differentiation of SH-SY5Y cells was with all-trans Retinoic Acid (RA, 20 µM, Sigma). For pharmacological studies 3-Methyladenine (3MA, Sigma-Aldrich), NH_4_Cl (Sigma-Aldrich), Bafilomycin (Baf, Sigma-Aldrich), alpha-methyl-p-tyrosine (AMPT, Sigma-Aldrich) and doxycyclin (dox, Clontech) were added at indicated times and concentrations.

### Primary neuronal cultures

Cultures of rat (embryonic day 18, E18) cortical neurons were prepared as previously described [56], [57]. Cells were plated onto poly–D-lysine-coated dishes at a density of approximately 150.000–200.000/cm^2^ and maintained in Neurobasal medium, with B27 supplement (Invitrogen), L-glutamine (0.5 mM) and penicillin/streptomycin (1%). More than 98% of the cells cultured under these conditions represent post-mitotic neurons [58].

### Intracellular protein degradation

Total protein degradation was measured by pulse-chase experiments as previously described [27]. Proteolysis was expressed as the percentage of the initial total acid-precipitable radioactivity (protein) in the cell lysates transformed to acid soluble radioactivity (amino acids and small peptides) in the medium during the incubation. Total lysosomal degradation was estimated as the NH_4_Cl- or Baf- inhibited degradation, while macroautophagic degradation was estimated as the 3MA-inhibited proteolysis.

### Cell Death

Cultures were exposed to Ethidium Homodimer (1 µM, Molecular Probes), which labels dead cells, and Hoechst 33342 (1 µM; Sigma), which labels cell nuclei. The percentage of dead cells, (Ethidium Homodimer-positive over total Hoechst-positive nuclei), was assessed in three separate wells, counting at least 100 cells per well.

For neuroprotection assays 3MA (10 mM, 36 hrs) or alpha-mehtyl-p-tyrosine (AMPT, 1 mM) were added 5 days after RA addition.

#### Assessment of survival

Viable cells were quantified by counting the number of intact nuclei in a haemocytometer, after lysing the cells in a detergent-containing solution [59], [60]. This method has been shown to be reproducible and accurate and to correlate well with other methods of assessing cell survival-death [56], [61]. Cell counts were performed in triplicate and are reported as means±SE.

### Generation of Recombinant Adenovirus

The recombinant adenoviral vectors expressing various forms of ASYN were constructed using the AdEasy system as previously described [62].

For primary cell infections, viruses (MOI 150) were added to primary cortical cultures 5 days after plating.

### RNAi

Small interfering RNAs were designed against human and rat ATG 5 mRNAs according to the criteria of Elbashir *et al* [63] and Reynolds *et al* [64]. The nucleotide sequences targeting ATG 5 were: 5′-AACCTTTGGCCTAAGAAGAAA-3′ (human) and 5′-AAGTCAGGTGATCAACGAAAT-3′ (rat). As controls, all Star Negative Control siRNA (#1027281, Qiagen) and a scrambled (scr) siRNA containing the sequence 5′-AACGAGAAGCAGAGCCATACT-3′ were used respectively.

siRNAs at a concentration of 25 nM were delivered with Lipofectamine 2000 (Invitrogen) to differentiated SH-SY5Y or PC12 cells. ATG 5 down-regulation was assessed 72 h post-transfection. Cortical neurons were infected with the human A53T ASYN adenovirus for 24 hours. The next day, the medium was removed and neurons were transfected with the rat ATG 5 or the scr siRNA together with EGFP plasmid (to monitor transfection efficiency) for 6 hours. 72 hours post-transfection, cells were treated with Ethidium Homodimer (1 µM, 15 min, 37°C) in combination with Hoechst 33342 (1 µM, Sigma). Neurons were fixed in 3.7% formaldehyde and death was estimated by assessing the percentage of EGFP/ATG 5 or EGFP/scr siRNA positive (green) cells which were Ethidium Homodimer-positive (red). To verify that the rat ATG 5 siRNA is functional in cortical neurons, we have used a GFP-LC3 plasmid that allows monitoring of LC3 I to II conversion (kind gift of Tamotsu Yoshimori, National Institute of Genetics, Japan). Cortical neurons were co-transfected with the scr or the ATG 5 siRNA along with the GFP-LC3 construct. Twenty four hrs post-transfection, autophagy was induced by addition of rapamycin (500 nM, 48 h) and cultures were observed under a fluorescent microscope for the formation of autophagic vacuoles.

### Western immunoblotting

Western blotting was performed as described previously [27]. Membranes were probed with antibodies against: *1*) ASYN, polyclonal C20 (1∶1000; Santa Cruz), 2) polyclonal ERK (1∶5000; Santa Cruz), 3) polyclonal LC3 (1∶1000, Molecular Probes), 4) polyclonal ATG 5 (1∶1000, Sigma). Blots were probed with HRP-conjugated secondary antibodies (Jackson). Intensity of immunoreactive bands was estimated by densitometric quantification using the Gel analyzer v1.0 software.

### Statistical analysis

All data are expressed as mean±SE. Statistical significance of differences was evaluated either with Student's t-test or with one way ANOVA followed by the Student-Newman-Keuls' test. Probability values <5% were considered significant.

## Supporting Information

Figure S1 WT or mutant ASYN-induction in cycling PC12 or SH-SY5Y cell lines doesn't affect cell survival. Proliferating PC12 (A) or SH-SY5Y (B) cells were induced to express (−dox) WT or mutant ASYNs for 10 days and then stained with Propidium Iodide (1 µM), which labels dying cells. Cell nuclei were counterstained with the Hoechst 33342 dye. Quantification of the percentage of dying cells stained with Propidium Iodide compared to the total number of Hoechst-positive nuclei is depicted. At least 100 Hoechst-positive cells were counted per well per condition. All presented data are the mean of 3 independent experiments and within each experiment triplicate samples per condition were assessed.
(1.64 MB TIF)

Figure S2 Inhibition of macroautophagy increases human over-expressed WT and A53T ASYN levels in differentiated SH-SY5Y cells. SH-SY5Y cells expressing WT or A53T ASYN were differentiated with 20 µM Retinoic Acid (RA) for 5 days in the absence of dox. 3MA (10 mM) was added to the cultures for 36 hrs. Untreated cells were used as controls (ctrl). Cell lysates were assessed by western immunoblotting for ASYN levels. ERK Ab was used as a loading control. (A, B) Representative immunoblots of ASYN levels in WT (A) and A53T (B) expressing cells. (C) Quantification of WT or A53T ASYN levels after 3MA addition, compared to controls. Results are expressed as the ratio of OD values to the corresponding controls and data are presented as the mean±S.E. of 3 independent experiments (**p<0.01, student's t-test comparing 3MA treated cells with the controls).
(2.08 MB TIF)

Figure S3 Down-regulation of human ATG 5 in differentiated WT and A53T ASYN expressing SH-SY5Y cells. SH-SY5Y cells expressing WT or A53T ASYN were differentiated with 20 µM Retinoic Acid (RA) for 5 days in the absence of dox and then transfected with ATG 5 or scrambled (scr) siRNA. Seventy two hrs later, cells were lysed and assayed for ATG 5 expression. ERK Ab was used as a loading control. (A, B) Representative immunoblots of ATG 5 levels in WT (A) and A53T (B) expressing cells. (C) Quantification of ATG 5 levels in cells transfected with ATG 5 compared to cells transfected with scr siRNA. Results are expressed as the ratio of OD values to the corresponding controls and data are presented as mean of ±S.E. of 3 independent experiments (*p<0.05, student's t-test comparing ATG 5 siRNA with the control scr siRNA treated cells).
(2.09 MB DOC)

Figure S4 Over-expression of A53T ASYN in primary cortical neurons causes CMA dysfunction. Rate of CMA-dependent [the difference between NH4Cl (total lysosomal) and 3MA (macroautophagy)-dependent] long lived protein degradation in rat cortical cultures, 96 hrs after transduction with adenoviruses expressing A53T or ΔDQ/A53T ASYN. EGFP transduced neurons are used as controls. All presented data are the mean of 4 independent experiments and within each experiment triplicate samples per condition were assessed (*p<0.05, one way ANOVA followed by the Student-Newman-Keuls' test, comparing between cultures expressing A53T ASYN and control EGFP; #p<0.05, comparing between cultures transduced with A53T and ΔDQ/A53T ASYN).
(1.42 MB TIF)

Figure S5 Down-regulation of rat ATG 5 in PC12 cells suppresses macroautophagy induction in the presence of rapamycin. Naive PC12 cells were transfected with ATG 5 or scrambled (scr) siRNA. Forty eight hrs after siRNA transfection rapamycin (rap, 500 nM) was added to the cultures. Seventy two hrs post-transfection, cells were lysed and assayed for ATG 5 and LC 3 levels. ERK Ab was used as a loading control. Representative immunoblots of ATG 5 and LC 3 in the presence or absence of rapamycin (rap) are presented in the upper panel. ATG 5 and LC 3 II levels in cells transfected with ATG 5 were compared to cells transfected with scr siRNA. Results are expressed as the ratio of OD values to the corresponding controls and data are presented as mean of ±S.E. of 4 independent experiments (*p<0.05, **p<0.01, one way ANOVA followed by the Student-Newman-Keuls' test, comparing ATG 5 levels in ATG 5 siRNA with the control scr siRNA-treated cells; #p<0.05, comparing LC3 II levels in ATG 5 siRNA with the control scr siRNA treated cells+/−rapamycin).
(2.66 MB TIF)
