# Supplementary figures and images for: Abberant α-Synuclein Confers Toxicity to Neurons in Part through Inhibition of Chaperone-Mediated Autophagy
Source: PLoS One. 2009 May 13;4(5):e5515. doi: 10.1371/journal.pone.0005515 (PMC2677735; doi:10.1371/journal.pone.0005515)

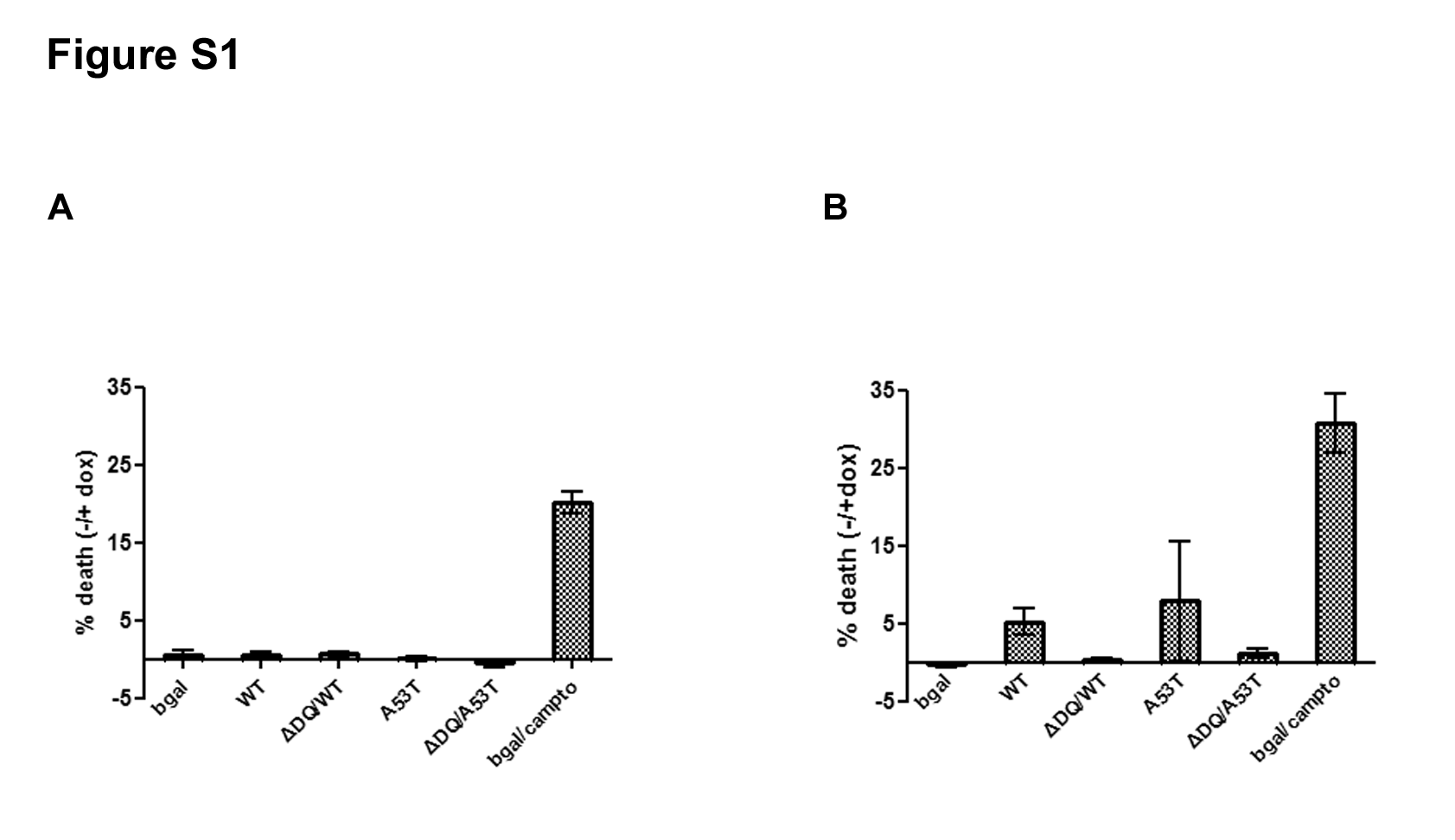

Supplement: Figure S1 — WT or mutant ASYN-induction in cycling PC12 or SH-SY5Y cell lines doesn't affect cell survival. Proliferating PC12 (A) or SH-SY5Y (B) cells were induced to express (−dox) WT or mutant ASYNs for 10 days and then stained with Propidium Iodide (1 µM), which labels dying cells. Cell nuclei were counterstained with the Hoechst 33342 dye. Quantification of the percentage of dying cells stained with Propidium Iodide compared to the total number of Hoechst-positive nuclei is depicted. At least 100 Hoechst-positive cells were counted per well per condition. All presented data are the mean of 3 independent experiments and within each experiment triplicate samples per condition were assessed. (1.64 MB TIF) [file pone.0005515.s001.tif]

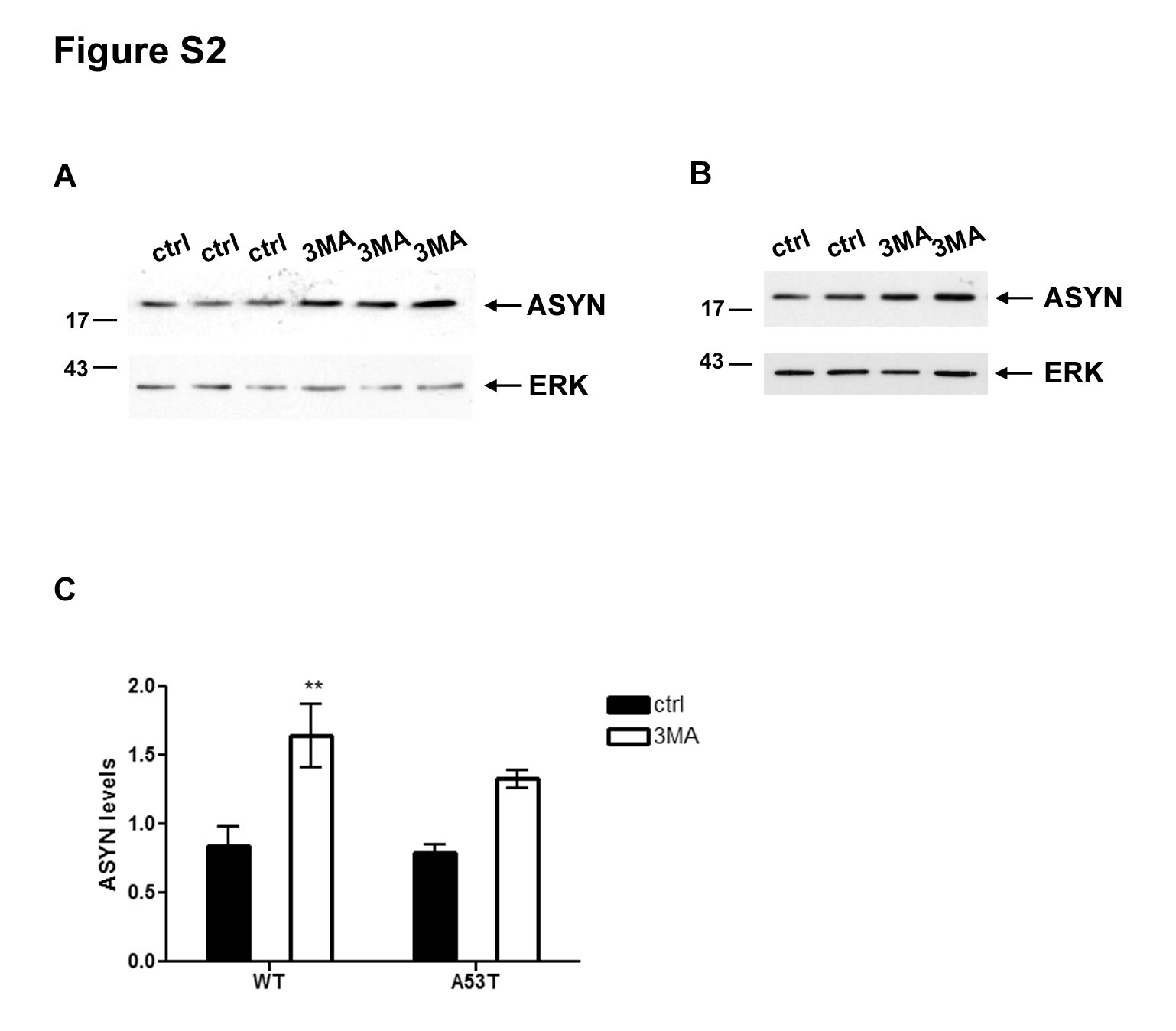

Supplement: Figure S2 — Inhibition of macroautophagy increases human over-expressed WT and A53T ASYN levels in differentiated SH-SY5Y cells. SH-SY5Y cells expressing WT or A53T ASYN were differentiated with 20 µM Retinoic Acid (RA) for 5 days in the absence of dox. 3MA (10 mM) was added to the cultures for 36 hrs. Untreated cells were used as controls (ctrl). Cell lysates were assessed by western immunoblotting for ASYN levels. ERK Ab was used as a loading control. (A, B) Representative immunoblots of ASYN levels in WT (A) and A53T (B) expressing cells. (C) Quantification of WT or A53T ASYN levels after 3MA addition, compared to controls. Results are expressed as the ratio of OD values to the corresponding controls and data are presented as the mean±S.E. of 3 independent experiments (**p<0.01, student's t-test comparing 3MA treated cells with the controls). (2.08 MB TIF) [file pone.0005515.s002.tif]

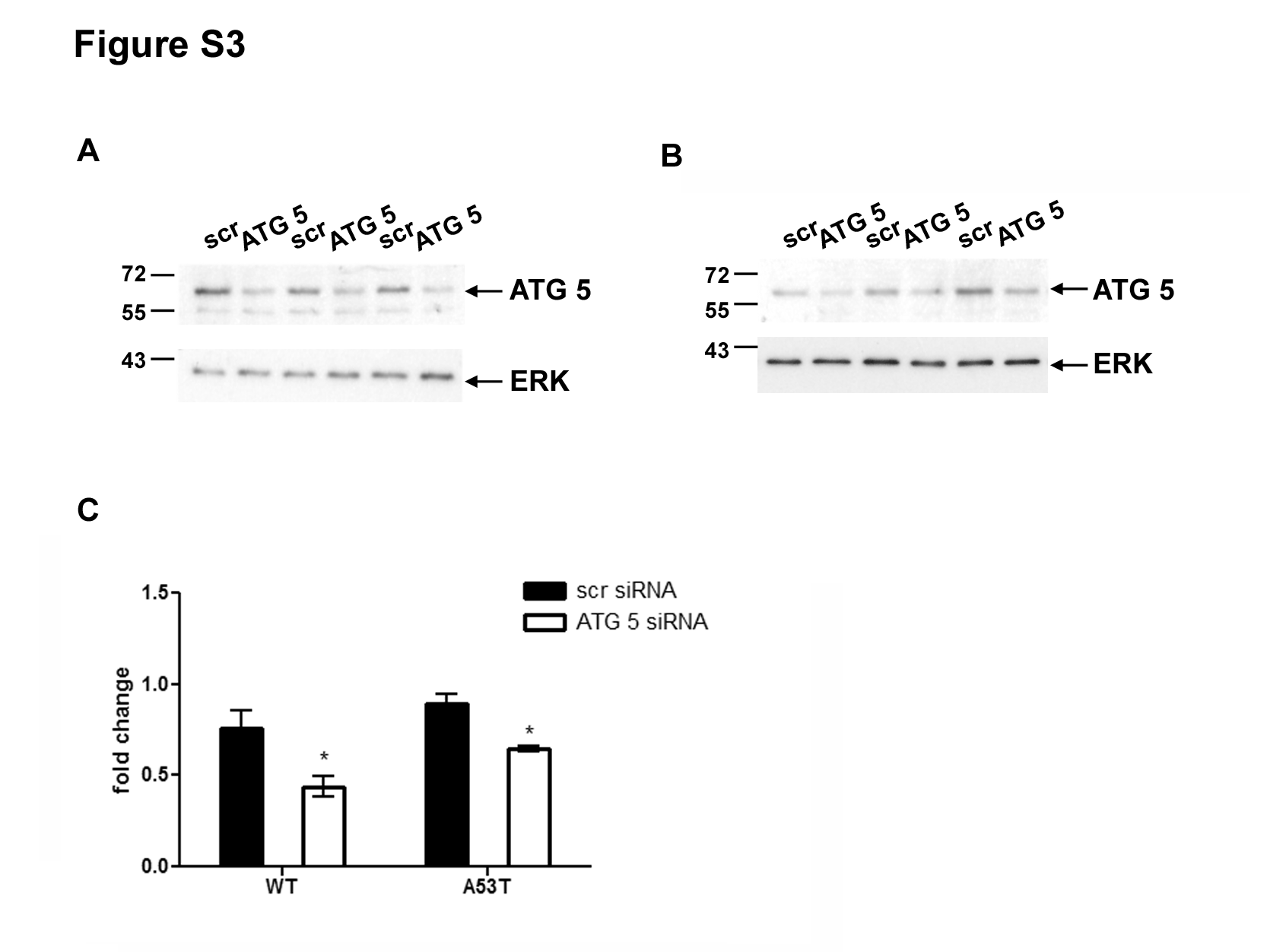

Supplement: Figure S3 — Down-regulation of human ATG 5 in differentiated WT and A53T ASYN expressing SH-SY5Y cells. SH-SY5Y cells expressing WT or A53T ASYN were differentiated with 20 µM Retinoic Acid (RA) for 5 days in the absence of dox and then transfected with ATG 5 or scrambled (scr) siRNA. Seventy two hrs later, cells were lysed and assayed for ATG 5 expression. ERK Ab was used as a loading control. (A, B) Representative immunoblots of ATG 5 levels in WT (A) and A53T (B) expressing cells. (C) Quantification of ATG 5 levels in cells transfected with ATG 5 compared to cells transfected with scr siRNA. Results are expressed as the ratio of OD values to the corresponding controls and data are presented as mean of ±S.E. of 3 independent experiments (*p<0.05, student's t-test comparing ATG 5 siRNA with the control scr siRNA treated cells). (2.09 MB DOC) [file pone.0005515.s003.tif]

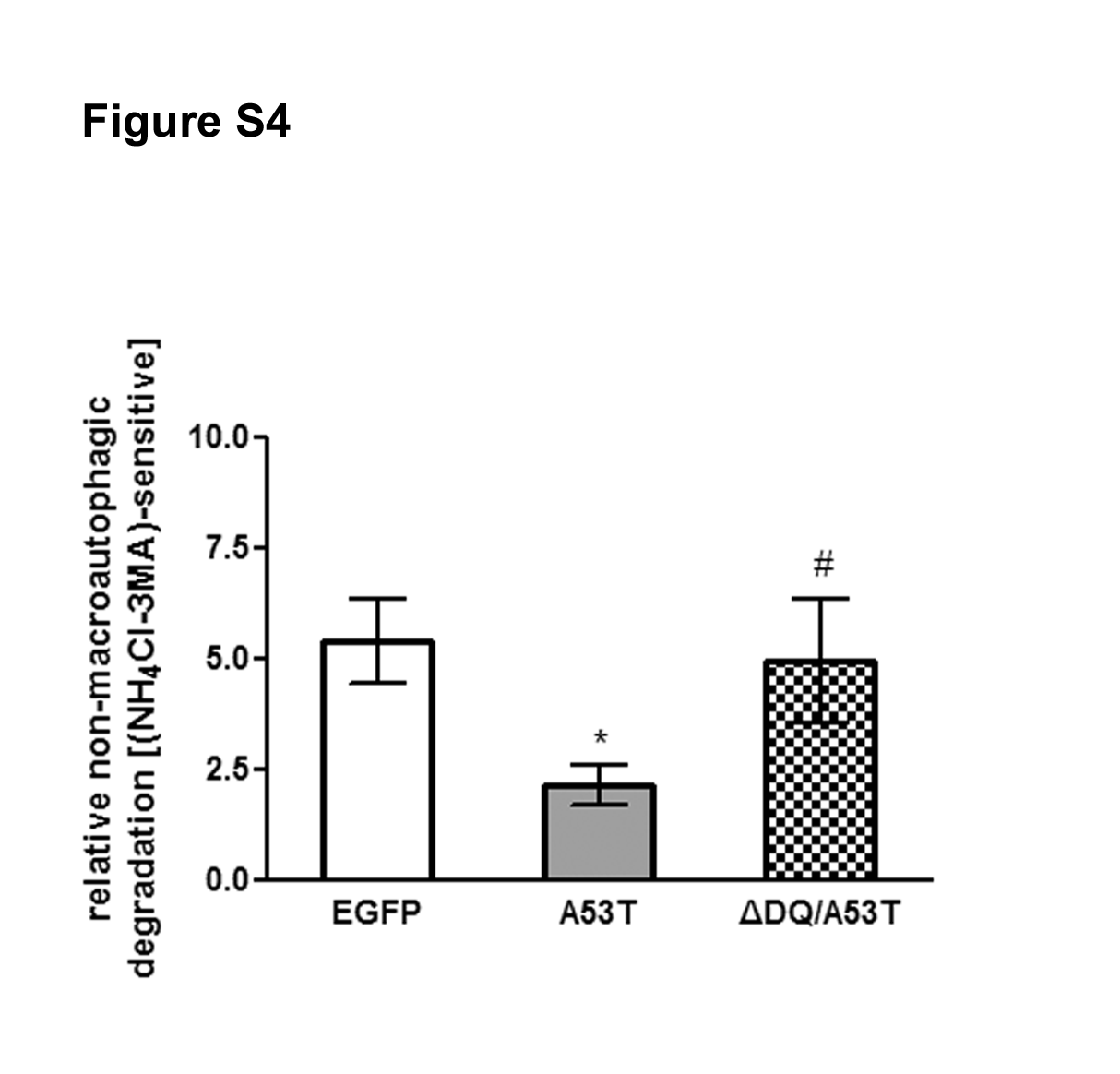

Supplement: Figure S4 — Over-expression of A53T ASYN in primary cortical neurons causes CMA dysfunction. Rate of CMA-dependent [the difference between NH4Cl (total lysosomal) and 3MA (macroautophagy)-dependent] long lived protein degradation in rat cortical cultures, 96 hrs after transduction with adenoviruses expressing A53T or ΔDQ/A53T ASYN. EGFP transduced neurons are used as controls. All presented data are the mean of 4 independent experiments and within each experiment triplicate samples per condition were assessed (*p<0.05, one way ANOVA followed by the Student-Newman-Keuls' test, comparing between cultures expressing A53T ASYN and control EGFP; #p<0.05, comparing between cultures transduced with A53T and ΔDQ/A53T ASYN). (1.42 MB TIF) [file pone.0005515.s004.tif]

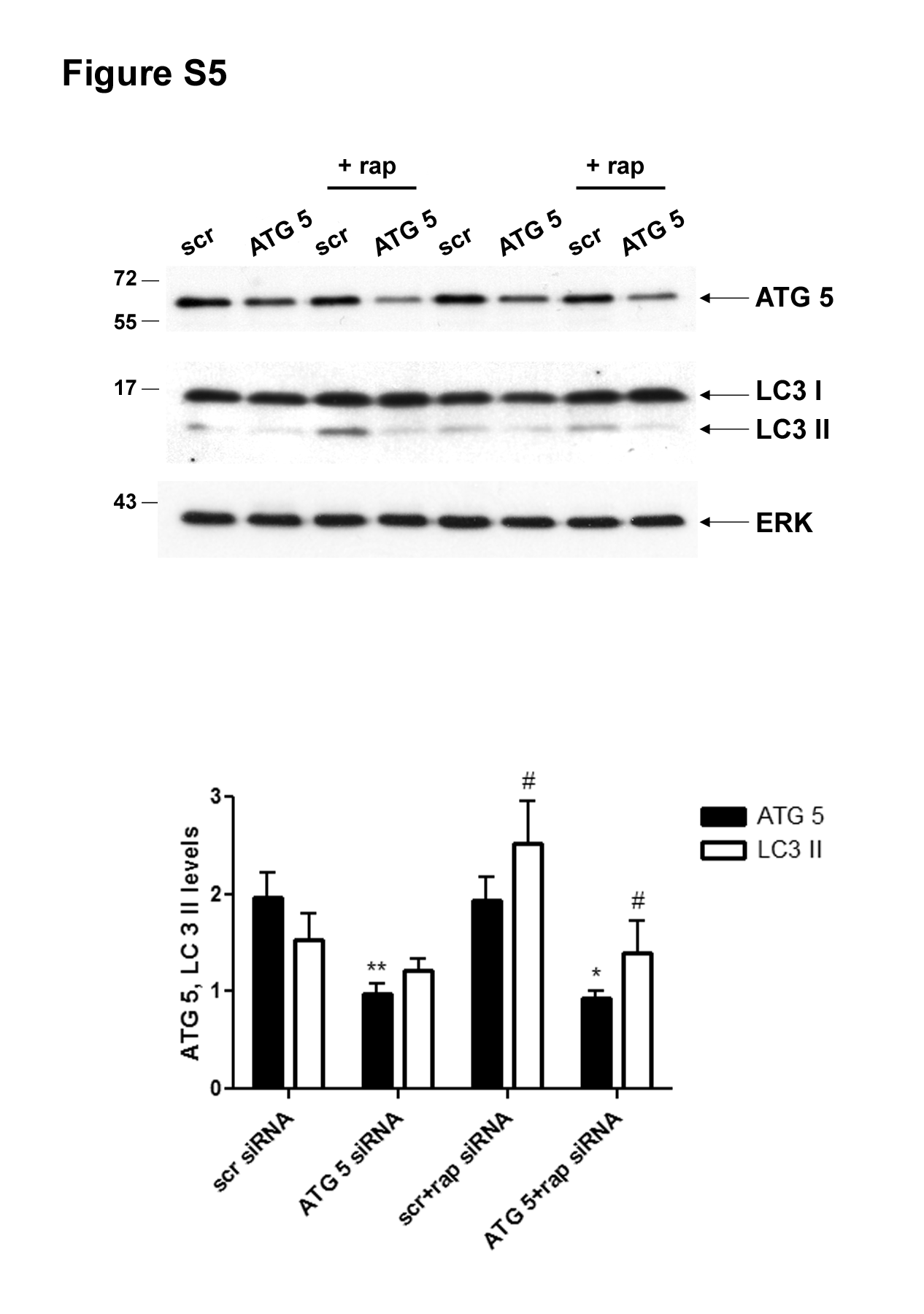

Supplement: Figure S5 — Down-regulation of rat ATG 5 in PC12 cells suppresses macroautophagy induction in the presence of rapamycin. Naive PC12 cells were transfected with ATG 5 or scrambled (scr) siRNA. Forty eight hrs after siRNA transfection rapamycin (rap, 500 nM) was added to the cultures. Seventy two hrs post-transfection, cells were lysed and assayed for ATG 5 and LC 3 levels. ERK Ab was used as a loading control. Representative immunoblots of ATG 5 and LC 3 in the presence or absence of rapamycin (rap) are presented in the upper panel. ATG 5 and LC 3 II levels in cells transfected with ATG 5 were compared to cells transfected with scr siRNA. Results are expressed as the ratio of OD values to the corresponding controls and data are presented as mean of ±S.E. of 4 independent experiments (*p<0.05, **p<0.01, one way ANOVA followed by the Student-Newman-Keuls' test, comparing ATG 5 levels in ATG 5 siRNA with the control scr siRNA-treated cells; #p<0.05, comparing LC3 II levels in ATG 5 siRNA with the control scr siRNA treated cells+/−rapamycin). (2.66 MB TIF) [file pone.0005515.s005.tif]
